# Health Mode On and Health Mode On Plus: a multisite protocol to assess and promote mental wellbeing and healthy lifestyles among Italian university students

**DOI:** 10.3389/fpubh.2026.1786142

**Published:** 2026-07-21

**Authors:** Giacomo Pietro Vigezzi, Giansanto Mosconi, Giovanni Albini, Silvio Brusaferro, Anna Carli, Michele Emdin, Fabrizio Faggiano, Anna Ogliari, Alfredo Raglio, Caterina Rizzo, Emanuele Rossi, Anna Odone, Health Mode On Investigators, Giovanni Albini, Chiara Barbati, Lavinia Barone, Mariagrazia a Baroni, Paola Bertuccio, Laura Brunelli, Silvio Brusaferro, Anna Carli, Virginia Casigliani, Cristiano Crescentini, Elisa Diadema, Michele Emdin, Fabrizio Faggiano, Daniele Fedeli, Andrea Fossati, Emina Mehanović, Giansanto Mosconi, Anna Ogliari, Anna Odone, Francesca Pennucci, Filippo Quattrone, Alfredo Raglio, Caterina Rizzo, Emanuele Rossi, Valentina Tobia, Riccardo Vecchio, Giacomo Pietro Vigezzi, Federica Vigna-Taglianti, Elena Vivaldi, Luca Viviani, Patrizia Zeppegno, Marzia Zingarelli

**Affiliations:** 1 Department of Public Health, Experimental and Forensic Medicine, University of Pavia, Pavia, Italy; 2 Conservatory of Music Antonio Vivaldi, Alessandria, Italy; 3 Department of Medicine, University of Udine, Udine, Italy; 4 Accreditation, Quality, and Clinical Risk Unit, Friuli Centrale Healthcare University Trust, Udine, Italy; 5 Conservatory of Music Rinaldo Franci, Siena, Italy; 6 Interdisciplinary Research Center for Health Science, Sant’Anna School of Advanced Studies, Pisa, Italy; 7 Department of Sustainable Development and Ecological Transition, University of Eastern Piedmont, Vercelli, Italy; 8 Osservatorio Epidemiologico, ASL Vercelli, Vercelli, Italy; 9 Faculty of Psychology, Vita-Salute San Raffaele University, Milan, Italy; 10 Child in Mind Lab, Vita-Salute San Raffaele University, Milan, Italy; 11 IRCCS San Raffaele Hospital, Milan, Italy; 12 Conservatory of Music Giuseppe Verdi, Milan, Italy; 13 Istituti Clinici Scientifici Maugeri IRCCS, Pavia, Italy; 14 Department of Translational Research and of New Surgical and Medical Technologies, University of Pisa, Pisa, Italy; 15 Conservatory of Music Pietro Mascagni, Livorno, Italy; 16 Sant’Anna School of Advanced Studies, Pisa, Italy; 17 Medical Direction, Fondazione IRCCS Policlinico San Matteo, Pavia, Italy

**Keywords:** addictive behaviors, longitudinal studies, mental health, university students, digital mental health

## Abstract

**Introduction:**

University is a pivotal life-course transition in which academic, social and geographic changes intersect with heightened risk for mental disorders and maladaptive behaviors. Health Mode On (HMO) and its longitudinal expansion Health Mode On Plus (HMO+), funded by the Italian Ministry of University and Research (MUR) competitive PRO-BEN 1 and PRO-BEN 2 calls, couple harmonized surveillance with a stepped, integrated model of prevention and care aligned with the World Mental Health–International College Student (WMH-ICS) framework to address fragmentation of campus provision and unmet need.

**Methods and analysis:**

HMO implements a cross-sectional, census-style online survey paired with the implementation of an integrated counseling pathway and a Virtual Academy for staff and student mentors. HMO+ adds 12-month re-contact, objective lifestyle sub-studies, and a strengthened shared digital platform that supports first-contact intake, triage, and orientation to the most appropriate service. Within this framework, students with an anxiety risk profile may be offered participation in an embedded two-arm randomized evaluation of digital cognitive-behavioral therapy (e-CBT) versus counseling-as-usual. The consortium spans five universities, four higher artistic and musical education (AFAM) institutes and one Scuola Superiore Universitaria, in coordination with University Sports Centres (CUS). Primary outcomes are 12-month and lifetime DSM-5 disorder status derived from validated WMH-ICS/CIDI-based modules (mood; anxiety; trauma- and stressor-related; obsessive-compulsive and related; eating; attention-deficit/hyperactivity; and substance use disorders), and safety outcomes (suicidal ideation, planning and non-suicidal self-injury). Secondary outcomes include psychological distress, role impairment, days-out-of-role, sleep, physical activity (accelerometry in subsamples), dietary habits, nicotine and cannabis use, behavioral addictions (e.g., gaming, gambling, internet addiction), social connectedness, academic engagement and service access. Analyses will estimate weighted prevalence and impairment with multilevel models; longitudinal change with mixed-effects models and GEE; and naturalistic program effects using difference-in-differences with propensity-score methods.

**Ethics and dissemination:**

Ethics approvals will be obtained at the coordinating and partner institutions. GDPR-compliant governance and results’ FAIR-oriented sharing are planned, alongside open-access publications, institutional dashboards and policy briefs.

## Introduction

University represents a critical life course transition in which academic, social and geographical changes reshape students’ daily routine, identity, health and help-seeking behaviors. Epidemiological studies consistently show that the first onsets of common mental disorders cluster before age 25, with anxiety and impulse-control disorders typically emerging in adolescence and mood and substance use disorders in early adulthood (1, 2). In university populations, cross-national evidence from the World Mental Health–International College Student (WMH-ICS) initiative indicates substantial prevalence and impairment alongside wide cross-institutional variability and unmet treatment need (3–6). Parallel reviews suggest that, while students report symptoms at least as frequently as non-students of the same age, service uptake remains limited and often delayed because of attitudinal (e.g., stigma, perceived norms) and structural (i.e., wait-times, availability) barriers (7–10).

These challenges have intensified in the past decade, with repeated cross-sectional surveys and administrative series documenting sustained demand that outpaces the capacity of traditional campus counseling models (10). The COVID-19 pandemic further amplified stressors and, in many settings, increased the prevalence of depressive and anxiety symptoms among university populations (11, 12). Within a behavioral epidemiology framework, mental health in emerging adulthood is tightly interwoven with modifiable lifestyle risks and digital habits. For instance, sleep disturbance prospectively predicts depression onset, with meta-analytic evidence showing approximately a twofold risk among people with insomnia compared with those without sleep difficulties (13). Physical activity is inversely associated with incident depression across age groups, with protective effects observed at relatively modest doses in prospective cohorts (14). Diet quality has also been implicated: adherence to healthy dietary patterns is inversely associated with depressive outcomes in longitudinal syntheses (15).

In the risk domain, harmful alcohol use and behavioral addictions, including problematic gaming, social media and gambling, are salient in student populations and are now measurable with brief, validated instruments (IGDS9-SF, BSMAS, PGSI) (16–19). The integration of these tools within a coherent measurement architecture allows the study of subjective symptoms and objective lifestyle indicators and, in turn, enables stratified prevention strategies targeting the most modifiable risk patterns.

Therefore, to address fragmentation across counseling units, inclusion services, sports centers and territorial providers, Health Mode On (HMO) project and its follow-up expansion, Health Mode On Plus (HMO+), couple rigorous surveillance with a stepped, multidisciplinary intervention model.

## Objectives

The program pursues a unified set of aims across assessment, intervention, training, communication and dissemination, and monitoring and evaluation. HMO aims to: (i) estimate point prevalence and determinants of psychological distress and risk behaviors; (ii) codesign and implement a locally adaptable model of integrated counseling linked to territorial specialist services; and (iii) establish a Virtual Academy to upskill staff and student tutors. HMO+ aims to consolidate and expand this model by conducting a 12-month individual follow-up, strengthening the e-counseling platform and triage, embedding objective measures of physical activity and diet, and testing a brief, digitally delivered Cognitive-Behavioral Therapy (CBT) protocol for anxiety via a randomized comparison that will be registered and reported separately. The overarching objective is to generate actionable evidence for scalable, equitable campus mental-health systems. Across HMO and HMO+, assessment is intended not only to describe prevalence and determinants, but also to inform service refinement, triage, and targeted intervention within a learning-system framework.

This protocol is therefore theorized within an ecological risk-and-resilience framework. At the individual level, the program supports autonomy, competence, and relatedness, cultivates emotion-regulation skills, and addresses modifiable habits (e.g., sleep, movement, diet, substance, and digital use). At interpersonal and meso-system levels, it enhances social connectedness and peer support as determinants of help-seeking and recovery (8, 9). At the system level, methodological alignment with WMH-ICS secures international comparability and benchmarking (3, 4). At the same time, the longitudinal design clarifies temporal dynamics and heterogeneity of response, key prerequisites for learning systems and equitable scale-up.

## Methods

### Setting

The consortium comprises five universities-Università degli Studi di Pavia (coordinating center), Università di Pisa, Università Vita-Salute San Raffaele, Università degli Studi di Udine, and Università degli Studi del Piemonte Orientale - together with the Scuola Superiore Sant’Anna (Pisa) and four AFAM institutions: Conservatorio di Musica “Giuseppe Verdi” di Milano, Conservatorio “Antonio Vivaldi” di Alessandria, Istituto Superiore di Studi Musicali “Pietro Mascagni” di Livorno and, in the second funding phases, Istituto Superiore di Studi Musicali “Rinaldo Franci” di Siena. Operationally, activities unfold within three territorial university–AFAM dyads: Lombardy (Pavia and Vita-Salute San Raffaele with the Milan Conservatory), Piedmont (Piemonte Orientale with the Alessandria Conservatory), Tuscany (Pisa and Scuola Superiore Sant’Anna with the Livorno Institute), each working in close coordination with the local Centro Universitario Sportivo (CUS) and territorial mental-health services. Program governance and standard operating procedures are harmonized to ensure comparability and fidelity while permitting local adaptation.

### Study design

**Figure 1:**
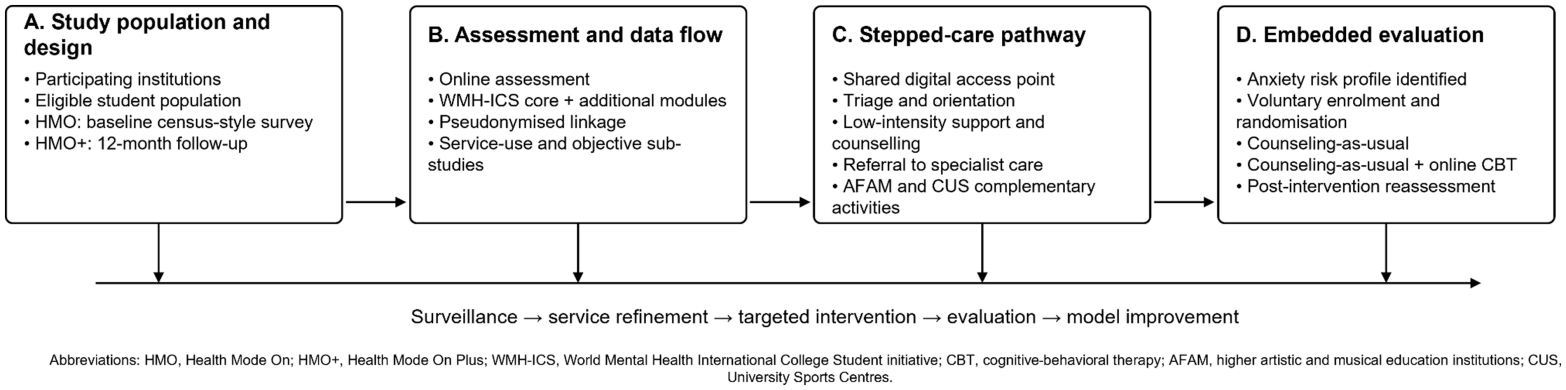
Overall architecture of the Health Mode On and Health Mode On Plus program: study design, data flow, intervention pathway, and embedded randomized evaluation.

HMO adopts a cross-sectional, census-style online survey of the eligible student population in each partner institution, paired with the implementation of an integrated counseling, sports- and music-supported intervention model and structured training for personnel and students. HMO+ adds a longitudinal component, re-contacting consenting HMO participants at 12 months while refreshing baseline cohorts with first-year enrollees to stabilize age-structure over time. The overall design combines surveillance, service implementation, and evaluation within an iterative learning-system approach. A schematic overview of the program architecture, including study design, data flow, intervention pathway, and embedded randomized evaluation, is provided in Figure 1.

### Study population and eligibility

The target population comprises all enrolled undergraduate and postgraduate students at participating universities and AFAM conservatories. Eligibility requires current enrollment, age ≥18 years, and provision of electronic informed consent; there are no exclusions related to health status or field of study. Recruitment leverages institutional mailing lists and learning platforms, amplified by student associations and institutional channels. Given the census-style design across multiple and heterogeneous institutions, no single response-rate target was prespecified. To mitigate non-response and attrition, the survey is modular and concise, staged reminders are deployed, and communications emphasize benefits and confidentiality. Mitigation of non-response bias therefore relies not only on weighting at the analysis stage, but also on recruitment design, institutional dissemination, student engagement, and a questionnaire structure intended to support participation and completion.

For HMO+ re-contact, a secure linkage mechanism generates salted hash-based keys from stable contact fields stored in a restricted vault, enabling longitudinal follow-up without storing direct identifiers in analytic files, in line with GDPR (20). Longitudinal analyses will use all available repeated observations, while attrition will be explicitly acknowledged as a potential source of bias in interpretation.

### Interventions and implementation strategy

The implementation arm builds a stepped, integrated counseling pathway that strengthens existing university services, formalizes interfaces with second-level territorial mental-health and addiction services, and connects with AFAM conservatories and University Sports Centers (CUS) to embed music- and sport-based programs as health-promoting adjuncts. Delivery modalities include in-person and tele-counseling coordinated through a shared digital platform that offers first-contact intake, orientation and triage to the most appropriate service. Within this stepped-care pathway, students are oriented according to their assessment profile and presenting needs. Possible pathways include university counseling services, disability and Specific Learning Disabilities (SLD), and neurodevelopment support, nutrition services, and referral to second-level territorial specialist care when more complex conditions emerge. Across sites, implementation is standardized through a shared consortium framework, common procedures, and harmonized training, while preserving flexibility for local adaptation to local institutional and territorial service configurations.

A Virtual Academy provides competency-based training for staff and student mentors through a training-of-trainers (ToT) model and co-designed psychoeducational workshops. Students participate in governance through a standing committee to promote acceptability and reduce stigma-related barriers to care, as documented in prior research (8). The observational component is intended to directly inform the intervention component. Assessment data are used to identify risk and protective profiles, unmet needs, and patterns of service access, and in HMO+ they also support refinement of the counseling model and targeting of at-risk students through the shared digital platform. Longitudinal and comparative analyses are also intended to provide feedback for ongoing strengthening of the intervention model over time.

Within HMO+, a randomized evaluation will test the added value of a brief, digital CBT protocol for students screening positive for clinically significant anxiety, compared with counseling-as-usual. Participants will be randomly assigned to counseling-as-usual plus 10 online CBT sessions or counseling-as-usual alone. In this context, counseling-as-usual refers to the routine counseling pathway available across participating sites and accessed through the integrated intake and triage system. The primary outcomes (change in anxiety severity at 10–12 weeks) will be collected with self-administered instruments and automated scoring. The trial will be prospectively registered and reported under CONSORT to ensure transparency and replicability (21, 22), and some implementation details of the trial, including delivery platform and adherence metrics, will be fully prespecified in the dedicated RCT protocol. The choice of digital CBT is supported by meta-analytic and randomized evidence in university samples (23–25).

### Outcomes

Primary outcomes align with the WMH-ICS instrument and comprise 12-month and lifetime DSM-5 disorder status derived from validated screening and diagnostic modules covering mood disorders (major depressive episode; bipolar spectrum), anxiety disorders (generalized anxiety disorder, panic disorder, social anxiety disorder, agoraphobia), trauma- and stressor-related disorders (post-traumatic stress disorder), obsessive-compulsive and related disorders, eating disorders (anorexia nervosa, bulimia nervosa, binge-eating disorder), attention-deficit/hyperactivity disorder and substance use disorders (alcohol, cannabis and other drugs). Suicidal ideation, suicide planning and non-suicidal self-injury are treated as primary safety outcomes. WMH-ICS relies on CIDI-based and CIDI-SC screening scales with established operating characteristics, and applies appropriate survey weights in disorder and impairment estimation (3, 26, 27).

Secondary outcomes include psychological distress and wellbeing, role impairment and days-out-of-role, sleep quality and disturbance, physical activity and sedentary time (self-report and accelerometry in a subsample), dietary quality, nicotine and cannabis use, behavioral addictions (problematic internet/social media use, gaming and gambling), social connectedness, academic engagement and performance proxies, service utilization, waiting times, referral completion and satisfaction, enabling evaluation of reach, equity and patient-centeredness (16, 18, 19, 28–33).

### Measures and data collection

Mental health self-report outcomes are collected using WMH-ICS baseline and 12-month follow-up instruments in their Italian version, consistent with DSM-5 clinical diagnostic criteria, to ensure international comparability (3, 4).

Relevant exposures and covariates are investigated through validated standard scales: the selection and integration of the specific tools within the WMH-ICS instruments is guided by an eDelphi process and is described elsewhere (34). As an example, sleep is measured with the Pittsburgh Sleep Quality Index (PSQI) (12, 28); physical activity is assessed with the International Physical Activity Questionnaire-Short Form (IPAQ-SF) and, in a subsample, with wrist-worn accelerometers to derive activity and sleep–wake parameters using established wear/non-wear algorithms (29). Tobacco and cannabis use, problematic internet and social media use, gaming and gambling are captured with brief, internationally used screeners such as the IGDS9-SF for gaming disorder, the Bergen Social Media Addiction Scale and the Problem Gambling Severity Index, leveraging available Italian adaptations (18, 19, 30). Contextual covariates include housing status, socioeconomic indicators, disability and SLD supports, academic engagement and use of counseling or specialist services. Additional measures were selected on the basis of validation status and the availability of Italian versions or adaptations where available. Although the protocol spans multiple domains, the questionnaire was structured to remain modular and concise in order to support feasibility at scale and reduce respondent burden.

### Data management

Data collection uses secure institutional platforms with encryption at rest and in transit. Personally identifying information is stored separately from survey responses and sensor data, linked by pseudonymous keys; linkage for HMO+ follows a salted-hash mechanism within a restricted re-contact vault. Datasets are version-controlled and fully documented, with a public codebook and de-identified aggregates released in line with the FAIR data principles and institutional policy (35). Processing complies with GDPR and applicable national regulations, with data sharing governed by data-transfer agreements and explicit participant consent (20).

### Sample size and power

The observational components are designed as a census of the eligible student population across sites (i.e., partners), maximizing precision of prevalence estimates and enabling multilevel modeling of determinants and heterogeneity. For the embedded trial, planning targets a minimum detectable standardized mean difference of approximately 0.30 in anxiety severity at 90% power and *α* = 0.05, implying roughly 234 participants per arm. These figures will be recalibrated if early variance or intra-individual correlations materially diverge from assumptions. The observational and experimental components serve different inferential purposes; accordingly, formal power considerations are presented for the embedded randomized evaluation, whereas the census-style observational component is designed primarily to maximize precision and support multilevel modeling of heterogeneity across sites.

### Statistical analysis

Survey data will undergo robust descriptive analysis with post-stratification or raking weights where appropriate to reduce non-response bias. Cross-sectional associations will be modeled using multivariable generalized linear or additive models with institution-level random effects. Longitudinal analyses in HMO+ will use linear and generalized mixed-effects models and generalized estimating equations to estimate within-person change and to test interactions by subgroup and exposure to program components. Naturalistic evaluations of service exposure will combine difference-in-differences estimators with propensity-score methods (e.g., matching, stratification or inverse probability weighting) to mitigate confounding by indication (36, 37). These approaches are intended to strengthen causal inference in the observational evaluation of service exposure, but do not eliminate the possibility of residual bias. They rely on adequate overlap and conditional exchangeability with respect to measured covariates and, for difference-in-differences analyses, on the plausibility of parallel trends. Unmeasured confounding cannot be fully excluded; therefore, causal interpretation will remain cautious. Sensitivity analyses will be prespecified in the time-stamped Statistical Analysis Plan to assess robustness to alternative specifications. All primary and secondary analyses will be prespecified in a time-stamped Statistical Analysis Plan deposited before unblinding.

### Monitoring and quality assurance

Fidelity to the stepped-care model is monitored through structured checklists, activity logs and periodic audits. The Virtual Academy integrates competency assessments to verify skills acquisition among staff and peer mentors. A logic-model-based monitoring and evaluation framework specifies indicators for reach, dose, compliance, outcomes and equity, reviewed annually by an International Advisory Board with expertise in public mental health, epidemiology, clinical psychology, sports medicine and music therapy; feedback loops inform iterative optimization. These procedures are intended not only to monitor fidelity to the stepped-care model across sites, but also to support iterative optimization of triage, referral pathways, and service integration over time.

### Governance and oversight

The coordinating university provides scientific leadership, data architecture and development of the integrated counseling model. Partner universities contribute expertise in epidemiology, public health, and clinical and educational psychology; AFAM conservatories lead music-based components; and CUS co-lead sport-based programs. Student representation is institutionalized through a standing committee that is engaged across design, implementation, and dissemination. Governance structures are intended to support harmonization across sites while preserving the flexibility needed for local implementation.

### Patient and public involvement

Students contributed to the co-design of instrument wording, psychoeducational workshops and communication strategies through representative committees embedded at each site. They informed outcome selection, acceptable survey burden and referral pathways; they will receive plain-language summaries and be invited to comment on interpretation and dissemination materials before final reporting.

## Ethics and dissemination

### Ethics, safety, and risk management

In accordance with the Declaration of Helsinki, the protocol and amendments are reviewed by the coordinating center’s Ethics Committee and, where required, by local committees at partner institutions. Participation is voluntary with electronic informed consent. The survey provides automated guidance and immediate signposting for participants disclosing self-harm or suicidality, with optional rapid referral pathways co-developed with institutional services. The embedded trial follows a proportionate adverse-event monitoring plan for low-risk psychosocial interventions.

### Dissemination and knowledge translation

The program embraces open science through prospective registration of the observational cohort and the embedded trial, publication of the protocol, and sharing of de-identified aggregate data and code where permissible. Dissemination to leadership, student bodies and national stakeholders will occur via institutional website,^1^ dashboards, policy briefs and workshops. Alignment with WMH-ICS supports international benchmarking and transferability across foreign campuses.

### Data sharing and availability

Upon completion of baseline and follow‑up waves, de‑identified aggregated data and analysis code will be shared in a public repository subject to institutional policy and participant consent; the persistent link will be provided in subsequent reports.

### Registration

The program is funded under the national call for promoting student mental wellbeing and countering psychological and emotional distress (PRO-BEN 1 and 2). The funders have no role in study design; data collection, management, analysis or interpretation; writing; or the decision to submit for publication. The observational cohort will be prospectively registered (e.g., ISRCTN), and the embedded trial will be registered on ClinicalTrials.gov (clinical trial number: not applicable); the Statistical Analysis Plan will be deposited on the Open Science Framework.

## Discussion

This protocol responds to a sustained increase in mental-health need among university students by embedding surveillance and implementation within a coherent stepped system of care. Evidence from systematic reviews indicates that digital mental-health interventions, including internet- and app-based cognitive-behavioral therapies, can improve depression and anxiety in student samples, with effects comparable to those observed in broader adult populations, although heterogeneity remains (23, 24). In resource-constrained campus settings, stepped and blended-care models are a pragmatic response to demand that outstrips capacity; longstanding guidance has recommended staff-to-student ratios that many institutions struggle to meet, contributing to wait-times and access bottlenecks (38). By deploying low-intensity digital supports as first-line options with clear pathways to higher-intensity care, HMO and HMO+ aim to increase reach and timeliness while maintaining fidelity to evidence-based practice.

The program also foregrounds modifiable behavioral determinants. Prospective meta-analyses suggest that sufficient physical activity, good quality sleep and healthy dietary patterns are inversely associated with depressive outcomes (13–15). By combining validated symptom scales with sleep, activity and diet measures, including a subsample with accelerometry and photographic food logging, the project enables the development of stratified prevention strategies that target the most flexible risk profiles in a student population.

Finally, the design leverages the WMH-ICS framework for international comparability, allowing benchmarking across institutions and countries and enabling pooled analyses in future collaborative work. This alignment, together with longitudinal follow-up in HMO+, supports the development of a learning system that can iteratively refine triage rules, outreach strategies and referral pathways. Anticipated limitations include selection and attrition biases in voluntary e-surveys and reliance on self-report for sensitive behaviors; weighting, rigorous follow-up procedures and triangulation with objective measures will mitigate residual bias, while transparent reporting and open-science practices will facilitate critical appraisal and replication. Additional limitations should also be acknowledged. First, the breadth of the web-based assessment may introduce respondent fatigue despite the modular design. Second, counseling-as-usual may retain some heterogeneity across sites, even within a shared consortium framework. Third, the naturalistic evaluation of service exposure remains vulnerable to residual confounding despite the use of propensity-score and difference-in-differences approaches. Finally, some implementation details of the embedded trial will be fully specified only in the dedicated RCT protocol and registration documents.

## Conclusion

Health Mode On and Health Mode On Plus operationalize a comprehensive, evidence-informed and scalable approach to student wellbeing that unites surveillance, implementation and rigorous evaluation. By combining a stepped, integrated care model with longitudinal epidemiology and targeted experimentation under international reporting standards and sound data governance, the program is positioned to generate actionable insights for universities in Italy and beyond and to provide a transferable template for sustainable institutional transformation.

## Footnotes

1
https://healthmodeon.it/

## Group members of Health Mode On Investigators

Giovanni Albini (Conservatory of Music Antonio Vivaldi, Alessandria), Chiara Barbati (Department of Public Health, Experimental and Forensic Medicine, University of Pavia, Pavia), Lavinia Barone (Department of Brain and Behavioral Sciences, University of Pavia, Pavia), Mariagrazia a Baroni (Conservatory of Music Antonio Vivaldi, Alessandria), Paola Bertuccio (Department of Public Health, Experimental and Forensic Medicine, University of Pavia, Pavia), Laura Brunelli (Department of Medicine, University of Udine, Udine; Accreditation, Quality, and Clinical Risk Unit, Friuli Centrale Healthcare University Trust, Udine), Silvio Brusaferro (Department of Medicine, University of Udine, Udine, Italy; Accreditation, Quality, and Clinical Risk Unit, Friuli Centrale Healthcare University Trust, Udine), Anna Carli (Conservatory of Music Rinaldo Franci, Siena), Virginia Casigliani (Department of Translational Research and of New Surgical and Medical Technologies, University of Pisa, Pisa), Cristiano Crescentini (Department of Languages and Literatures, Communication, Education and Society, University of Udine, Udine), Elisa Diadema (Department of Translational Research and of New Surgical and Medical Technologies, University of Pisa, Pisa), Michele Emdin (Interdisciplinary Research Center for Health Science, Sant’Anna School of Advanced Studies, Pisa), Fabrizio Faggiano (Department of Sustainable Development and Ecological Transition, University of Eastern Piedmont, Vercelli, Italy; Osservatorio Epidemiologico, ASL Vercelli, Vercelli), Daniele Fedeli (Department of Languages and Literatures, Communication, Education and Society, University of Udine, Udine), Andrea Fossati (School of Psychology, Vita-Salute San Raffaele University, Milan; Clinical Psychology and Psychotherapy Unit, San Raffaele Turro Hospital, Milan), Emina Mehanović (Department of Sustainable Development and Ecological Transition, University of Eastern Piedmont, Vercelli), Giansanto Mosconi (Department of Public Health, Experimental and Forensic Medicine, University of Pavia, Pavia), Anna Ogliari (Faculty of Psychology, Vita‐Salute San Raffaele University, Milan, Italy; Child in Mind Lab, Vita‐Salute San Raffaele University, Milan, Italy; IRCCS San Raffaele Hospital, Milan, Italy), Anna Odone (Department of Public Health, Experimental and Forensic Medicine, University of Pavia, Pavia; Medical Direction, Fondazione IRCCS Policlinico San Matteo, Pavia), Francesca Pennucci (Interdisciplinary Research Center for Health Science, Sant’Anna School of Advanced Studies, Pisa), Filippo Quattrone (Interdisciplinary Research Center for Health Science, Sant’Anna School of Advanced Studies, Pisa), Alfredo Raglio (Conservatory of Music Giuseppe Verdi, Milan; Istituti Clinici Scientifici Maugeri IRCCS, Pavia), Caterina Rizzo (Department of Translational Research and of New Surgical and Medical Technologies, University of Pisa, Pisa), Emanuele Rossi (Conservatory of Music Pietro Mascagni, Livorno; Sant’Anna School of Advanced Studies, Pisa), Valentina Tobia (School of Psychology, Vita-Salute San Raffaele University, Milan), Riccardo Vecchio (Department of Public Health, Experimental and Forensic Medicine, University of Pavia, Pavia), Giacomo Pietro Vigezzi (Department of Public Health, Experimental and Forensic Medicine, University of Pavia, Pavia), Federica Vigna-Taglianti (Department of Translational Medicine, University of Eastern Piedmont, Novara, Italy), Elena Vivaldi (Interdisciplinary Research Center for Health Science and Dirpolis Institute, Sant’Anna School of Advanced Studies, Pisa), Luca Viviani (Department of Public Health, Experimental and Forensic Medicine, University of Pavia, Pavia), Patrizia Zeppegno (Department of Translational Medicine, University of Eastern Piedmont, Novara, Italy, SC Psichiatria AOU Maggiore della Carità, Novara), Marzia Zingarelli (Conservatory of Music Antonio Vivaldi, Alessandria).
